# Developmental Origins of Health and Disease: A Lifecourse Approach to the Prevention of Non-Communicable Diseases

**DOI:** 10.3390/healthcare5010014

**Published:** 2017-03-08

**Authors:** Janis Baird, Chandni Jacob, Mary Barker, Caroline H. D. Fall, Mark Hanson, Nicholas C. Harvey, Hazel M. Inskip, Kalyanaraman Kumaran, Cyrus Cooper

**Affiliations:** 1 Medical Research Council Lifecourse Epidemiology Unit, University of Southampton, Southampton SO16 6YD, UK; meb@mrc.soton.ac.uk (M.B.); chdf@mrc.soton.ac.uk (C.H.D.F.); nch@mrc.soton.ac.uk (N.C.H.); hmi@mrc.soton.ac.uk (H.M.I.); kk@mrc.soton.ac.uk (K.K.); cc@mrc.soton.ac.uk (C.C.); 2 Institute of Developmental Sciences, University of Southampton, Southampton S016 5YA, UK; chandni.jacob@gmail.com (C.J.); M.Hanson@soton.ac.uk (M.H.); 3 National Institute for Health Research Southampton Biomedical Research Centre, University Hospital Southampton, NHS Foundation Trust, Southampton SO16 6YD, UK

**Keywords:** lifecourse, NCDs (non-communicable diseases), prevention, intervention

## Abstract

Non-communicable diseases (NCDs), such as cardiovascular disease and osteoporosis, affect individuals in all countries worldwide. Given the very high worldwide prevalence of NCDs across a range of human pathology, it is clear that traditional approaches targeting those at most risk in older adulthood will not efficiently ameliorate this growing burden. It will thus be essential to robustly identify determinants of NCDs across the entire lifecourse and, subsequently, appropriate interventions at every stage to reduce an individual’s risk of developing these conditions. A lifecourse approach has the potential to prevent NCDs, from before conception through fetal life, infancy, childhood, adolescence, adulthood and into older age. In this paper, we describe the origins of the lifecourse concept, the importance of early life influences, for example during pregnancy, examine potential underlying mechanisms in both cell biology and behavior change, and finally describe current efforts to develop interventions that take a lifecourse approach to NCD prevention. Two principal approaches to improving women’s nutritional status are outlined: nutritional supplementation and behavior change.

## 1. Introduction

Non-communicable diseases (NCDs) are responsible for a substantial burden of mortality and morbidity at a global level. They include cardiovascular disease, cancers, respiratory diseases, diabetes, obesity, and musculoskeletal disorders. Evidence from research carried out over the last thirty years has demonstrated that the environment during early life influences an individual’s risk of developing NCDs in later life. This evidence is the basis for the Developmental Origins of Health and Disease (DOHaD) concept. Following birth, environmental exposures during infancy, childhood and adult life can then further modify the risk of developing these chronic diseases in later life.

A lifecourse approach to NCD prevention is based on the premise that NCDs can be prevented and controlled at multiple stages of the lifecourse. The approach sees optimization of growth and development as being fundamental to the prevention of disease. Identifying and understanding the biological, behavioral and psychosocial processes that act during an individual’s lifecourse to influence their risk of disease gives rise to the potential for intervention in these pathways to ill health.

## 2. A Lifecourse Approach

Lifecourse epidemiology is the study of the long-term effects on later health and disease risk of physical or social exposures during gestation, childhood, adolescence, young adulthood, and later adult life. The aim of the approach is to find out about processes (biological, behavioral, and psychosocial) that operate across an individual’s lifecourse or across generations, to influence risk of disease.

The lifecourse approach is increasingly focused on the development and evaluation of interventions to improve health and prevent disease. Improved understanding of the mechanisms that underlie associations between early life and later disease is facilitating the development of interventions that can optimize growth and development of body composition, and maintain physical and cognitive function at all stages of childhood and adolescence.

The need to address risk factors for, and treatment of, NCDs across the whole of life, from before birth into older age, is supported by recent strategy from the World Health Organisation, the United Nations and the UK Department of Health [1,2].

**Figure 1:**
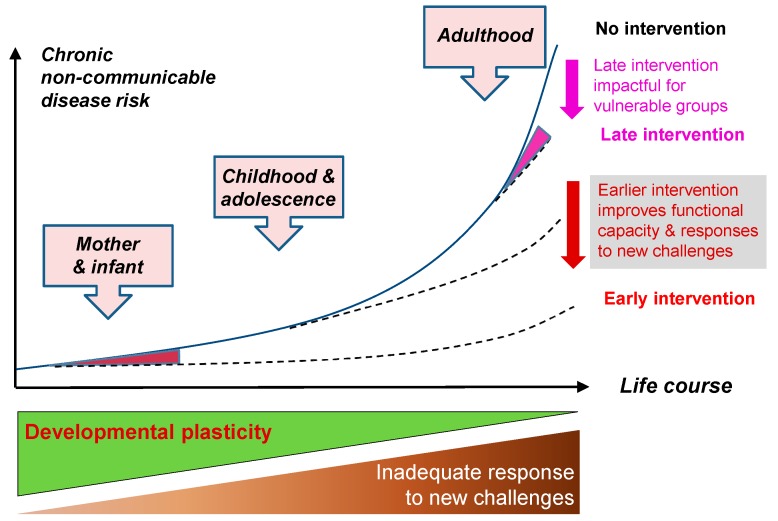
Timing of interventions and effect on disease risk. Reprinted from Hanson et al. [3] with permission.

An individual’s risk of developing NCDs is accumulated throughout their lifecourse. Figure 1 shows how risk increases as a result of declining plasticity (green triangle) and the resulting accumulated effects of inadequate responses to new challenges (brown triangle). The greatest increase in risk is acquired in adult life. However, risks begin to accumulate much earlier in life. Maternal factors such as diet and body composition will influence risk of disease before and during pregnancy. Fetal, infant and childhood nutrition and development will influence risk of disease thereafter. Taking a lifecourse approach enables early identification of phenotypes and markers of risk, and this in turn facilitates the development of nutritional and other lifestyle interventions aimed at preventing disease. Relatively modest interventions in early life (red area) can have a large effect on disease risk later (red arrow). Later intervention (pink area) can have an impact on disease risk for vulnerable groups (pink arrow). Early life preventive measures require a long term investment but can lead to large reductions in disease risk. Thus they are more likely to be effective at preventing NCDs than population screening programs that identify the early stages of disease only after the disease is already established.

## 3. Observational Evidence of a Link between Early Development and Later Disease

The last three decades has seen the emergence of evidence demonstrating the importance of the environment during early life for the establishment of disease risk in later life and in future generations. Studies in Hertfordshire, UK, were the first of a series of cohort studies that used historical records combined with later follow up, to explore the association of early life with chronic disease in adulthood. In Hertfordshire, sixteen thousand men and women born between 1911 and 1930 were traced. Death rates from coronary heart disease fell steadily across the birth weight distribution such that rates at the higher end of the distribution were roughly half those at the lower end [4]. Findings from the Swedish cohort study, which followed up 14,611 babies, also supported the inverse association between cardiovascular disease and birth weight [5]. These findings in relation to birth weight, as a marker of early life exposures, have been replicated in many cohorts in the UK and in Europe and for a number of chronic diseases including coronary heart disease, hypertension, stroke, type 2 diabetes, osteoporosis and sarcopenia. The associations of birth weight with these diseases were independent of lifestyle risk factors, including smoking and alcohol intake, and of socio-economic status.

The developmental origins model of disease pathogenesis is supported by biological evidence from animal experiments. These have shown that alteration of maternal diet during pregnancy can modify offspring physiological processes, and that these modifications are lasting rather than transient [6]. Such a phenomenon is an example of phenotypic plasticity where a genotype can give rise to different physiological or morphological states depending on the prevailing environmental conditions during development. Studies in experimental animals have made it clear that the long-term effects of early life nutrition act through developmental changes to organs and tissues such as the pancreas, liver, kidneys, skeletal muscle and adipose tissue. Newborn size (equivalent to birth weight in human studies) is frequently used as an indicator of the intra-uterine experience, because it is easy to measure, but can only be a crude proxy of these changes at tissue level.

Animal experiments have shown that overfeeding mothers with high fat or high energy diets, leading to maternal diabetes and obesity, will increase insulin resistance, diabetes and cardiovascular changes in their offspring [7,8]. Recently, there has been accumulating evidence that paternal diet, body composition and health can also affect the health of the offspring [9].

Multiple developmental factors operate from preconception through early life to affect the risk of later NCDs including obesity, type 2 diabetes, cardiovascular disease, bone and joint disease, respiratory diseases such as asthma and some forms of cancer, and so the potential for preventing NCDs by achieving optimal fetal development is now increasingly recognized [10]. Low birth weight, an indicator of poor nutrition in utero, is associated with higher infant mortality, poorer educational outcomes in childhood and poorer long term health [11]. Social, psychological and occupational exposures during infancy, childhood and adult life will modify risk of ill health and disease.

## 4. Maternal Nutrition

Observational evidence of a link between early life and later disease has led to an interest in maternal influences on the development of the fetus. A girl or woman’s nutritional status before and during pregnancy influences outcomes both for her pregnancy and for the developing fetus [12]. Poor maternal nutrition can increase the risk of adverse pregnancy outcomes. It also has a strong influence on risk of pre-term delivery and impaired growth and development in utero and after birth. These, in turn, can lead to long-term effects for the baby due to influences on physical and cognitive development during infancy and childhood and metabolic adaptations to poor nutrition that can influence risk of later NCDs including cardiovascular disease and obesity. Recent studies have shown that prenatal exposure to gestational diabetes could lead to epigenetic alterations that increase the risk of type 2 diabetes later in life. Evidence from low and middle-income countries (LMICs) has also supported these findings. In India, for example, findings of the Pune maternal nutrition study suggest that micronutrient deficiencies (such as vitamin B12) can also lead to low birth weight and an increased risk of later diabetes [13].

Maternal undernutrition is usually caused by food shortage or economic hardship which leads to food insecurity and result in inadequate intake of nutrients. Exposure to undernutrition in utero is associated with low birth weight and stunting in childhood, which are in turn associated with shorter adult height and reduced economic productivity [14,15]. Undernutrition in utero also has adverse effects on cognitive development and so is also associated with lower levels of educational attainment. Low birth weight, due to maternal undernutrition, has been associated with increased risk of NCDs and obesity in later life [16,17].

Maternal overnutrition can also be harmful for both mother and fetus. Overnutrition occurs when the energy consumed outstrips energy expended and usually leads to overweight and obesity. For women during pregnancy, overnutrition leads to greater risk of gestational diabetes and hypertensive disorders of pregnancy. For the fetus, maternal gestational diabetes leads to an increased risk of macrosomia, high blood glucose and insulin and these are associated with neonatal hypoglycemia, congenital anomalies, preterm birth, stillbirth and neonatal death. There is also evidence that obese women accumulate more metabolites in their ovarian follicles and this has been associated with increased risk of cardiovascular disease and obesity in later life in their offspring [18].

Deficiencies of specific vitamin and minerals can be caused by insufficient intake due to poor or inadequate diet, or by an increased demand for nutrients, for example because of rapid growth or menstrual bleeding. Micronutrient deficiencies can occur even when there is overnutrition, and lifestyle factors such as alcohol intake and smoking can affect their absorption. Deficiencies of vitamins and minerals in mothers will affect their offspring as many micronutrients pass across the placenta from mother to fetus. For example, maternal 25(OH)-vitamin D [25(OH)D] status influences offspring bone mass at birth. The Princess Anne study—a cohort study of pregnant women and their children in Southampton—demonstrated a positive relationship between maternal antenatal 25(OH)D and offspring bone mass: low maternal 25(OH)D in late pregnancy was associated with reduced whole body bone mineral content and bone mineral density of the offspring when assessed by dual-energy X-ray absorptiometry (DXA) at nine years of age [19]. Similar results have emerged from the Southampton Women’s Survey (at birth and six years) [20], with recent findings from the Australian Raine cohort demonstrating relationships persisting to 20 years old [21], around the age of peak bone mass [21].

There is increasing evidence that the effects of maternal diet on the later health of the fetus are determined before pregnancy begins, highlighting the importance of preconception as a period for prevention of later NCDs [22]. Many interventions to improve maternal nutrition begin only once a women knows she is pregnant and seeks ante-natal care, thus missing the majority of the first trimester, when placentation and organogenesis occur. Studies in Southampton have shown that women of childbearing age, who are disadvantaged by having low levels of educational attainment, have diets of poor quality [23]. Maternal diets of poor quality have been associated with less optimal patterns of skeletal development, adiposity and cognitive development in their children [24]. Evidence shows that many women (especially young women) do not plan or prepare for pregnancy and unplanned pregnancies are still common [22]. The health behaviors of women during pregnancy are strongly influenced by their social circumstances and studies have shown that only a small proportion of women planning a pregnancy follow the recommendations for a healthy pregnancy such as increased fruit and vegetable consumption, folic acid intake, smoking and alcohol cessation [25].

## 5. Mechanisms

The etiology of NCDs is complex and both genetic and environmental factors play a role. There is much interest in the scope for NCD prevention and treatment through the identification of candidate genes. Genetic polymorphisms could potentially explain both poor fetal development and later risk of disease. A study by the Wellcome Trust Care Control Consortium identified several new genetic loci and genes that influence an individual’s susceptibility to a range of conditions including coronary heart disease and type 1 and 2 diabetes [26]. However, while candidate genes have been identified for some NCDs, there is little evidence that these genes are also linked to fetal development. More importantly, even combining the effects of known genetic loci associated with particular diseases does not account for a substantial levels of risk at the population level [27].

### 5.1. Epigenetic Mechanisms

The emergence of epigenetics is allowing exploration of the molecular mechanisms that link early exposures to later disease. Epigenetic modification does not result from changes to the sequence of bases in DNA itself but from changes to gene expression, which is mediated by DNA methylation, chromatin modification or small non-coding RNAs in response to the environment in which the fetus develops. Epigenetic mechanisms underlie the developmental plasticity, that is fundamental to the link between fetal development and risk of later disease [3]. There is evidence that maternal factors can modulate gene expression in their offspring thus influencing [8,28]. For example maternal malnutrition had led to altered gene methylation and increased risk of offspring metabolic syndrome in adult life [29]. In addition, recent studies have shown that prenatal exposure to gestational diabetes could lead to epigenetic alterations that increase the risk of type 2 diabetes later in life [30]. Influences of early development on satiety and food preferences suggest that, once set points are established in early life, it may be difficult or even impossible to reverse them. This might explain why lifestyle interventions in adult can have limited effects and are difficult to sustain [22]. Animal studies have also shown that supplementation with folic acid during pregnancy can potentially prevent some of the epigenetic changes that underlie the development of NCDs [31].

### 5.2. Behavioural Mechanisms

The health behaviors that people adopt will modify their risk of disease across the lifecourse. Childhood and adolescence are stages of the lifecourse when health behaviors become established [32,33]. Smoking and tobacco use, unhealthy diet, physical inactivity and the harmful use of alcohol are all associated with increased risk of NCDs. These risk factors are responsible for considerable burden of disease on a global level [34]. They can have direct effects on health or can act by influencing the development of high blood pressure and elevated blood glucose and cholesterol levels, which will then raise the risk of chronic diseases such as cardiovascular disease and diabetes. The transition in health risks occurring in LMIC populations due to the decline in incidence of infectious disease, changing patterns of physical activity and diet, and an ageing population has led to a doubling of NCD prevalence over recent decades [35].

Risk factors for NCDs, including raised blood pressure, adverse lipid profiles and obesity, can begin to develop during childhood and then track into adolescence and adulthood. Establishing healthy eating and physical activity patterns during infancy childhood will promote health and protect against the development of NCDs across the lifecourse [32]. There is well established evidence of the benefits of breastfeeding in reducing the risk of infection, and protecting the infant from sudden infant death syndrome (SIDS). There is also evidence that infants who are breastfed have reduced risk of obesity and diabetes in adulthood. Poor diet is common during childhood including iron and vitamin deficiencies during infancy and consumption of inappropriate energy-dense foods that increase the risk of obesity during childhood [36,37]. The way in which parents feed their children and control what they eat has a strong influence on children’s early eating patterns and risk of childhood obesity, and physical activity and sedentary behaviors in parents are often mirrored in the behaviors of their children [32]. The potential for adoption of adverse health behaviors makes adolescence a key stage in the development of NCDs [33]. Adolescence is a period of physical and psychological change and a phase when young people develop independence. New behaviors developed during adolescence can have positive or negative consequences for health [38]. Behaviors like smoking and alcohol use developed during adolescence will track into adult life, highlighting the importance of intervening during this period to prevent later disease.

Pregnancy during adolescence is an important issue in both developed country settings and in the developing world. Pregnancy at a young age, and early marriage, not only affect the health and human rights of girls but also disrupts their education and development of skills and social networks, all of these undermining their future health and wellbeing, along with the health of their children [39]. Adolescent pregnancy is associated with higher risk of adverse outcomes for both mother and child than pregnancies occurring when women are aged 20–30 years; stillbirths, neonatal deaths, preterm births, low birth weight and postnatal depression are all more common in adolescent pregnancies [40,41]. Pregnancies occurring at a younger age are often unplanned and so risk factors for adverse pregnancy outcome, such as low folic acid intake and alcohol use, are more likely. These can have a detrimental effect on the early development of the fetus [42].

## 6. Interventions

The observational and mechanistic evidence demonstrating the influence of maternal nutrition on the future health of their offspring, has led to a strong focus on the improvement of the health and nutrition of women of childbearing age. Nutritional supplementation (multiple micronutrient supplementation, and single vitamin supplements to correct deficiencies) and behavior change offer two approaches to improving the nutritional status of women during preconception and pregnancy [42]. For the correction of micronutrient deficiencies, such as vitamin D deficiency during pregnancy, traditional randomized controlled trials provide robust, well-controlled frameworks for theoretical and pragmatic evaluation of the candidate policy. In evaluations of behavior change interventions more complex strategies are required, and different evaluative models (such as complex intervention studies or natural experiments) need to be applied.

### 6.1. Nutritional Supplementation

Trials of nutritional supplementation include single vitamin supplements and multiple micronutrient approaches. When vitamin deficiencies occur during pregnancy, supplementation has been shown to prevent adverse effects for pregnant women and their babies in both developed and LMIC settings [43]. The recent UK-based trial of vitamin D supplementation—Maternal Vitamin D Osteoporosis Study (MAVIDOS), tested the hypothesis that maternal antenatal vitamin D supplementation leads to increased offspring bone mineral content. Just over 1100 women were randomized to 1000 IU vitamin D3 (cholecalciferol) per day or matched placebo. Women took the supplements from 14 weeks gestation until the birth of their baby. The study was a double-blind design across three study centers (Southampton, Sheffield, Oxford) [20]. Although there was no difference between the supplemented and unsupplemented groups in offspring whole body bone mineral content (BMC), measured by DXA within two weeks after birth, this relationship was markedly modified by season of delivery. Thus, in a pre-specified analysis, amongst winter births, neonates delivered to mothers allocated vitamin D supplements had more than 0.5 SD greater whole body BMC than did neonates born to placebo mothers (*p =* 0.004); the interaction between season and treatment was also statistically significant (*p =* 0.04) [44]. This season-treatment interaction is consistent with the known seasonal variation in 25(OH)D concentrations [45], these being lowest in winter/spring and highest in summer/autumn in temperate northern hemisphere countries. Where births occur during winter months, the lowest background 25(OH)D concentrations coincide with the period of greatest calcium transfer from mother to fetus in the last trimester. Indeed although 25(OH)D concentrations amongst placebo mothers rose from early to late pregnancy in mothers who delivered in summer/autumn, they declined for spring, and particularly winter deliveries. In contrast, 25(OH)D concentrations in mothers allocated to vitamin D supplementation rose regardless of season of delivery. Removal by the supplement of the seasonal drop in 25(OH)D consequent on winter delivery may be key to the bone findings, and further mechanistic work is ongoing. Vitamin D supplementation appeared safe; these findings support current recommendations in UK and elsewhere, and may suggest the basis for more stratified approaches to vitamin D supplementation in pregnancy.

Multiple micronutrient approaches have been evaluated in LMICs. In Mumbai, India, for example, the effects of a food-based supplement on maternal and infant outcomes were assessed in the Mumbai Maternal Nutrition Project (MMNP) [46]. The intervention, in over 6000 women, was a daily snack. For women in the intervention group the snack was made from green leafy vegetables, fruit, and milk, whereas women in the control group received a snack made up of low-micronutrient vegetables such as potato and onion. Women took the snacks daily from 90 days or more before pregnancy until delivery, in addition to the usual diet. Of 6513 women randomly assigned to treatment vs. control diet, 2291 women became pregnant, 1962 women had live singleton newborns, and, of these births, 1360 newborns were measured. The intervention had a marked effect on the prevalence of gestational diabetes—halving rates in women in the intervention group compared with women in the control group. There was a reduction in the prevalence of low birth weight among mothers who were not underweight and who were supplemented for three months before conception (treatment 34% vs. controls 41% in a per protocol analysis). Whether or not these changes lead to a reduction in NCD risk for the offspring remains to be seen. A recent systematic review found no convincing evidence of long-term benefits on growth, blood pressure or cognitive function, of maternal multiple micronutrient supplements started during pregnancy [47], but no studies of micronutrient supplementation starting preconceptionally, such as the Mumbai trial, have achieved long enough follow-up yet to answer this question.

### 6.2. Health Behaviour Change Interventions

Behavior change approaches during preconception and pregnancy can improve women’s health behaviors. While nutrient supplementation addresses specific nutrient deficiencies, behavior change approaches can improve overall diet quality. Pregnancy is a period when women are more likely to improve their health behaviors. Thus, it is a time when unhealthy behaviors, such as smoking and poor diet, can be tackled and healthier behaviors promoted [48]. Changing the health behaviors of women preconceptionally is more challenging not least because this group of women might still be adolescents with little understanding of the influence of their own health on that of their babies.

Women’s confidence, or self-efficacy, that they can make such changes is an important determinant of whether they will improve their health behaviors. Low levels of self-efficacy are common among women from disadvantaged backgrounds and mean that women are less likely to have healthy diets [49]. Many studies have demonstrated a relationship between higher levels of self-efficacy and better dietary behaviors [50]. Reviews of evidence have shown that interventions with certain features are more likely to improve health behaviors for disadvantaged women. These include: providing information on risks and benefits of health behaviors; goal-setting; and continued support after the initial intervention [51,52]. The evidence indicates that there is a need for empowerment approaches that work by improving the self-efficacy of participants.

Evidence from trials during pregnancy also points to the effectiveness of behavior change approaches. Two recent UK trials suggest that interventions during pregnancy can successfully improve women’s health behaviors. In the UPBEAT and LIMIT trials, women were supported throughout their pregnancies by regular contact with health care workers. These interventions led to improvements in diet although they did not improve the primary outcomes of gestational diabetes and babies born large for gestational age [53,54]. Importantly, both interventions included goal setting as a component suggesting that empowerment approaches are likely to be more successful in bringing about behavior change.

An empowerment approach was applied to a community-based intervention in Southampton, UK, targeting women of childbearing age, who already had at least one child, while they were between pregnancies. The intervention, the Southampton Initiative for Health, aimed to improve the health behavior of women from disadvantaged backgrounds. The intervention was set in Sure Start Children’s Centres [55]. These Centres were developed to provide services and support for women with children aged under five years with an initial focus on serving areas of disadvantage. Sure Start staff members come into contact with women and their children attending the Centres. The staff members were trained in skills to support behavior change: Healthy Conversation Skills [55]. As a result of the training, staff changed the way they interacted with women, using open discovery questions, listening more than talking and empowering women to set goals. These changes were still apparent one year after training [56]. Evaluation showed that women who came into contact with trained staff had significantly smaller declines in their sense of control and self-efficacy than women in the control group, although an effect on diet was not observed [57]. Self-efficacy and sense of control are psychological factors known to be associated with diet quality among disadvantaged women. These findings suggest that the intervention could improve women’s health behaviors if it were delivered in a setting that allowed frequent contact between women and trained staff. Women access services during pregnancy, providing an opportunity for repeated exposure to the Healthy Conversation Skills intervention and a trial that is assessing the efficacy of the intervention during pregnancy in women who receive antenatal care in Southampton’s maternity hospital is currently underway.

Changing the health behaviors of women preconceptionally is more challenging but, arguably more important than pregnancy as a period for prevention of later disease. The recent recommendations by The International Federation of Gyanecology and Obstetrics (FIGO) state that adopting healthy habits prior to conception, leading to improvements in maternal nutrition, provides important benefits for the next generation [42]. One of the challenges is how to engage women in interventions preconceptionally and to find ways of sustaining their engagement in a way that is both acceptable and affordable. The behavior change skills (Healthy Conversation Skills) implemented in the Southampton Initiative for Health can be used by health and social care staff in a range of settings and have the potential to address the challenges of engaging women preconceptionally. The skills are easily-acquired and theory-based, and are designed for use in brief consultations, to support diet and lifestyle change. Engaging adolescents is likely to pose additional challenges since they are less likely than women of other ages to be in contact with routine health and social care. More novel ways of promoting preconception health are required for this group. In the UK, educational interventions have the potential to improve health behaviors among adolescents. The Lifelab intervention is an example of such an intervention. Teenagers aged 13–14 years, who attend Hampshire secondary schools, have three weeks of school lessons, supported by teacher professional development, and a visit to an educational facility in the local hospital. The aim of Lifelab is to improve young people’s health literacy and understanding of the long-term influences of their health behaviors on their subsequent health and that of their children [58].

Similar approaches are being used in less developed communities. In South Africa, for example, rates of obesity are high among adolescent girls leading to high rates of gestational diabetes and low birth weight. An intervention to reduce obesity among adolescent girls is being developed, that will use community health workers trained in behavior change techniques, to empower adolescent girls to improve their health behaviors [59]. 

Novel technologies also have potential for engaging adolescents in changing their health behaviors. The reach and accessibility of digital interventions, added to the fact that approximately 90% of 16 to 24 year olds in high income countries (HICs) own smartphones, make them an obvious mode of delivery of behavior change interventions for adolescents [60]. Such interventions are becoming increasingly common, and there is some evidence of effectiveness [61] though surprisingly little of this evidence concerns adolescence. The ubiquity of mobile phone technology across LMICs and HICs suggests that this is a platform for delivering low-cost, population-level diet and lifestyle improvement. The challenge that remains is to overcome the problems of low usage, attrition and small effect sizes which have so far characterized such interventions [62]. Interventions across the lifecourse, particularly those focusing on early life factors, may also produce economic benefits. The economic benefit of reducing low birth weight in low-income countries has been estimated at approximately USD580 for each infant who achieves a normal birth weight. The main gains resulted from improved labor productivity as well as from reduced morbidity and mortality [63]. A lifecourse approach with a focus on early years also has the potential to reduce health inequalities which in turn will produce further economic benefits [10]. Future interventional studies should collect economic data in order to incorporate appropriate analyses of cost-effectiveness.

## 7. Conclusions

Taking a lifecourse approach to NCDs allows prevention at early stages of the lifecourse when effects on later disease risk have the potential to be substantial. Observational and mechanistic evidence has demonstrated the importance of maternal nutrition, during preconception and pregnancy, as an influence on future offspring health and has also shed light on the mechanisms that link maternal nutrition to fetal and childhood growth and development. The evidence points to the importance of interventions that have the potential to improve maternal nutrition, using a range of nutritional and behavioral strategies targeted at women before and during pregnancy.
